# Peroneal Nerve Repair with Cross-Bridge Ladder Technique: Parallel End-to-Side Neurorrhaphies

**DOI:** 10.1055/s-0043-1768996

**Published:** 2023-05-23

**Authors:** Simon Ammanuel, Daniel Burkett, Jason J. Kim, Evalina S. Bond, Amgad S. Hanna

**Affiliations:** 1 Department of Neurological Surgery, University of Wisconsin School of Medicine and Public Health, Madison, Wisconsin, United States

**Keywords:** cross-bridge, foot drop, ladder technique, nerve repair, peroneal nerve

## Abstract

**Background** Multiple nerve transfer techniques are used to treat patients with nerve injuries when a primary repair is not possible. These techniques are categorized to end-to-end, end-to-side, and side-to-side neurorrhaphy. Our study aims to explore the utility of the cross-bridge ladder technique (H-shaped), which has shown promising results in animal models and probably underutilized clinically.

**Methods** Four patients with significant loss of ankle dorsiflexion were seen in the clinic and underwent evaluation, including electrodiagnostic studies. A cross-bridge ladder repair technique was used between the tibial nerve as the donor and the common peroneal nerve as the recipient via one or two nerve grafts coapted in parallel with end-to-side neurorrhaphies. Dorsiflexion strength was measured preoperatively using the Medical Research Council (MRC) grading system and at each postoperative follow-up appointment.

**Results** All four patients had suffered persistent and severe foot drop (MRC of 0) following trauma that had occurred between 6 and 15 months preoperatively. Three of the four patients improved to an MRC of 2 several months postoperatively. The last patient had an immediate improvement to an MRC of 2 by his first month and had a complete recovery of ankle dorsiflexion within 4 months from surgery.

**Conclusion** We demonstrate the utility and clinical outcomes of the cross-bridge ladder technique in patients with persistent and prolonged foot drop following trauma. Both early and late recovery were seen while all patients regained motor function, with some patients continuing to improve up to the most recent follow-up.

IRB Approval: Obtained 2013–1411-CP005

## Introduction

Common peroneal nerve (CPN) dysfunction is the most common nerve abnormality in the lower extremity, likely because of its superficial location as it courses around the fibular neck. Damage to the CPN results primarily in foot drop and related gait abnormalities that may impair patients' ability to walk unassisted and cause unpleasant paresthesias in the lower lateral leg and dorsal foot.

Current methods of treatment include both surgical and nonsurgical options. Nonsurgical management utilizes ankle bracing with an ankle-foot-orthosis (AFO) or a spring-loaded ankle brace to compensate for the loss of ankle dorsiflexion. This is a fast and cost-effective way to improve a patient's foot drop to avoid tripping and falling; however, braces are often uncomfortable for patients, require life-long use, and do not correct the underlying problem.

Surgical treatments include decompression of the nerve, excision of the damaged nerve sections followed by either a primary repair or an interposition graft, nerve transfer between the tibial nerve and the peroneal nerve, and tendon transfers.

Functional outcomes of nerve repair are thought to be influenced by several factors, including the age of the patient, severity of nerve damage, length of injured segment, mechanism of injury, the distance from the lesion to the end organ, and the time lapse between nerve injury and treatment. 1 2 3 4 5 6 The best clinical outcomes following nerve transection are achieved with primary nerve repair ideally within 3 days following the injury. However, this is only possible in limited clinical scenarios. Nerve injury and dysfunction in patients with proximal nerve injuries or patients presenting for delayed surgical repair continue to have poor outcomes. 7 8 A delay in treatment and its impact on outcomes has been of particular interest as an intervenable factor in care. It is thought that the poor outcomes in patients with delayed treatment or proximal injuries are because of chronic denervation of the end organs and loss of Schwann cells. These changes include muscle atrophy, declining numbers of Schwann cells in the distal nerve, and reduced secretion of neurotrophins from these Schwann cells. 1 9 10 11 12 13

Multiple nerve repair techniques are employed to treat patients with these injuries when a primary repair is impossible. Patients with significant loss of nerve length or in patients with delayed nerve repair, a nerve transfer or nerve graft is preferable as tension-free neurorrhaphy avoids compromise of the endoneurial blood supply and necrosis. 14

These techniques can be categorized by the type of neurorrhaphy they utilize and include: end-to-end, end-to-side, and side-to-side, either directly or with grafts. The most utilized and researched technique in nerve repair is end-to-end with or without a graft, followed by end-to-side transfers either via the distal recipient nerve stump or the proximal donor nerve stump (i.e., supercharging nerve transfer). 14 However, research regarding these different techniques has failed to demonstrate the consistent superiority of any technique. 14 15 16 17 Therefore, this article aims to explore the utility of the cross-bridge ladder technique (H-shaped), which has shown promising results in animal models and may be underutilized in clinical studies. This technique uses one or more nerve grafts placed in parallel between a donor and recipient nerve in an end-to-side fashion without transection of the donor or recipient nerves. While this technique has demonstrated promising results in animal models of delayed surgical repair, 7 8 the full impact of this technique in this challenging population has yet to be defined in humans.

## Methods

The study received approval from the Institutional Review Board and ethics committee.

Four patients with significant loss of ankle dorsiflexion were seen in the clinic and underwent appropriate evaluation. Electrodiagnostic studies (EDX) were obtained for all patients, and after nonsurgical etiologies were ruled out, patients with significant disabilities related to their foot drop were offered surgical exploration and potential repair. Intraoperative neuromonitoring was used to identify the location of the lesion along the CPN. A cross-bridge ladder repair technique was used between the tibial nerve as the donor and the CPN as the recipient via one or two nerve grafts coapted in parallel with end-to-side neurorrhaphy. Nerve grafts that can be used are either allograft or autograft from the sural nerve. Dorsiflexion strength was measured preoperatively using the Medical Research Council (MRC) grading system and at each postoperative follow-up visit.

### Cross-Bridge Ladder Surgical Technique (H-shaped) for Peroneal Nerve Dysfunction

**Fig. 1:**
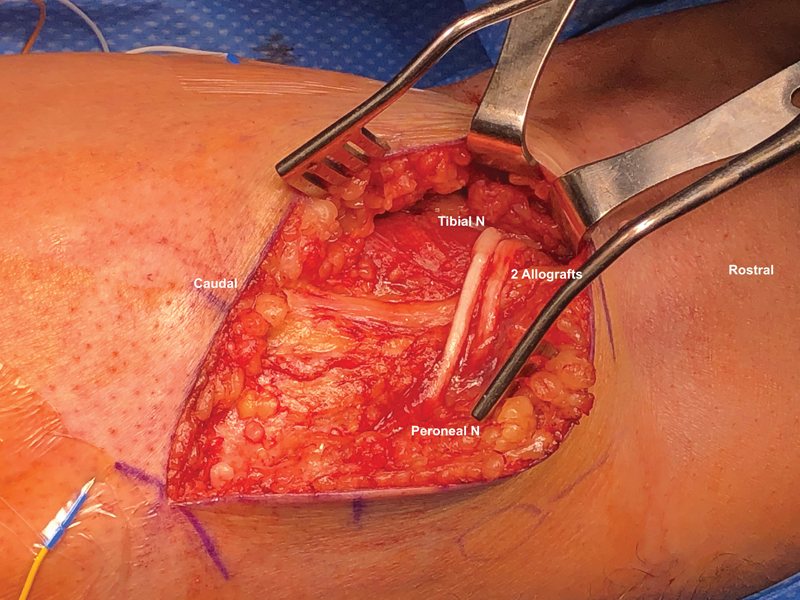
Image of the final cross-bridge transfer demonstrating the two allografts from the tibial nerve to the peroneal nerve with end-to-side coaptations.

Conceptually, this technique allows a functional side-to-side neurorrhaphy between two nerves through multiple nerve grafts coapted in parallel to the donor and recipient nerves in an end-to-side fashion ( Fig. 1 ). In the case of CPN dysfunction, exposure of the recipient CPN and donor tibial nerve is required. A skin incision is placed over the popliteal fossa to access both tibial and peroneal nerves through either a vertical or horizontal lazy-S incision. After sufficient decompression of the peroneal nerve is accomplished, perineural windows via small longitudinal incisions are created in the common peroneal and tibial nerves. The distance between the nerves is measured, and the appropriate grafts are cut to length, allowing a tension-free neurorrhaphy. The graft is buried in the incisions along the donor and recipient nerves and then secured in place with sutures and fibrin glue. After completing the first cross-bridge, this process can be repeated proximally or distally along both the common peroneal and tibial nerves with a second graft, creating a cross-bridging appearance of the coaptations ( Fig. 1 ). There was no crushing of the fascicles while attaching the end-to-side grafts. Grafts used could be allograft or autograft.

## Results

### Case 1

A 68-year-old man presented to clinic for a right-sided foot drop that had persisted since sustaining an injury due to a mechanical fall 4 months prior. He had been using a walker to assist with ambulation since then. He was also experiencing significant numbness and burning pain on the dorsal foot. His dorsiflexion MRC score was 0, while tibial nerve was 5. EDX evaluation showed likely sciatic neuropathy. Magnetic resonance imaging (MRI) of the knee shows evidence of subacute on chronic denervation of the muscles of the anterolateral compartment of the right leg. Otherwise, normal signal and course of visualized peroneal nerve and no visual evidence of compression near the fibular neck. The patient was taken to surgery for peroneal nerve release and cross-bridge nerve grafting. Intraoperative EDX shows no muscle movement when stimulating the CPN, while tibial nerve stimulation showed expected movements in soleus and gastrocnemius muscles. Two allografts were used as the cross-bridges in an end-to-side manner ( Fig. 1 ). At his 1-month follow-up, the patient had a dorsiflexion MRC of 0; then, at his 4-month follow-up, this had improved to an MRC of 2. EDX at his 4-month visit demonstrated improvement in peroneus longus and tibialis anterior muscle recruitment. The patient now walks using his AFO brace five times per week and 2 days per week walk unassisted. At 1-year follow-up, the patient was walking without his brace, and ankle dorsiflexion MRC improved to 4.

### Case 2

An 84-year-old woman presented to the clinic for a right-sided foot drop which began 14 months prior following right hip replacement surgery. Dorsiflexion MRC at the time was 0. She had decreased sensation along her dorsal foot. She was ambulating using an AFO brace. EDX evaluation demonstrated decreased amplitude and increased latency in the peroneal nerve. The patient was taken to the operating room (OR). Intraoperative EDX shows activation of tibial nerve with Nerve Action Potentials (NAPs) of 0.5 and movement of the soleus and gastrocnemius muscles, while no movement with CPN stimulation with as high as 20 milliamps. The peroneal nerve was decompressed, and one cross-bridging allograft was coapted between the tibial nerve and the peroneal nerve, end-to-side in an H-shaped manner. Her 1-month follow-up appointment MRC score was 0 but improved to 2 at her 3-month visit. Sensation over her dorsal foot remained decreased postoperatively.

### Case 3

A 23-year-old man presented to the clinic for left-sided foot drop, absent sensation, and neuropathic pain in the sural and peroneal nerve distributions 6 months after sustaining a gunshot wound in the left lower thigh. Ankle dorsiflexion MRC was 0 at the time. He required a walker for ambulation. EDX revealed no peroneal nerve function below the short head of the biceps femoris. Intraoperatively, both the tibial and peroneal nerves underwent release in the thigh and calf. Neuromonitoring showed positive responses throughout the peroneal and tibial nerves. MRI showed partial nerve injury superior to the bifurcation to tibial and peroneal nerves. Two allografts were used to bridge the tibial and peroneal nerves. At 1-month follow-up, the patient's ankle dorsiflexion was improved to 2, and at his 4-month follow-up, it had further improved to an MRC of 5. He has retained this at his most recent 8-month appointment. He has been ambulating unassisted since his 4-month appointment. His EDX at 4 months postoperatively demonstrated improved reinnervation of the tibial and peroneal nerve innervated muscles. Unfortunately, the patient developed severe pain in the CPN distribution, limiting his ability to work. At his 8-month follow-up, the patient had improved strength, but continued to have pain as preoperatively.

### Case 4

A 50-year-old male presented to the clinic for right foot drop and dorsal foot paresthesia sustained after fracture dislocation of his knee 15 months previously. Ankle dorsiflexion MRC was 0 and required an AFO brace for ambulation. Ankle plantar flexion MRC was 5. EDX revealed no motor units in the tibialis anterior and peroneus longus. Right leg MRI showed denervation atrophy of the anterolateral muscles. The patient was taken to the OR for release of the peroneal nerve and grafting with two allografts bridging parallel between the tibial and peroneal nerves. At his 1-, 4-, and 7-month follow-up appointments, the patient continued to have an ankle dorsiflexion MRC of 0; however, by his 1.5 years' follow-up, his MRC improved to 2, he still used an AFO brace for ambulation. At his 2- and 3-year follow-up appointments, his MRC has remained stable at 2, but he no longer wears his AFO brace for ambulation.

Overall, all four patients suffered persistent and severe foot drop (MRC of 0) following trauma that had occurred between 6 and 15 months preoperatively. Three of the four patients improved to an MRC of 2 over months to years postintervention. The third patient had an immediate improvement to an MRC of 2 by his first month and had complete recovery of ankle dorsiflexion within 4 months of surgery. While patients 1 and 2 are still being followed, their early improvement from an MRC of 0 preoperatively to an MRC of 2 within 4 months of surgery is promising. Notably, of the two patients who were followed for over 1 year, the one that reached full recovery dorsiflexion after just 4 months (patient 3) underwent surgery only 6 months after his initial injury, while the other (patient 4) did not receive surgical repair until 15 months after the initial trauma. None of the patients sustained additional deficits in the tibial nerve distribution postoperatively ( Table 1 ).

**Table 1:** Patient and surgical characteristics

	Age	Sex	Mechanism of injury	Style of graft	Time since injury	Preoperative walking status	Preop dorsiflexion MRC	Preoperative sensory function	Type and number of graft	Length of the graft(s)	Most recent follow-up	Most recent dorsiflexion MRC	Most recent walking status
Patient 1	68	M	Mechanical fall	End-to-side	6 mo	Assisted- walker	0	Absent in CPN distribution	Allograft ×2	N/A	1 y	4	Walks unassisted
Patient 2	84	F	Total hip replacement surgery	End-to-side	14 mo	Assisted- AFO brace	0	Decreased in CPN distribution	Allograft ×1	5 cm	3 mo	2	Assisted- AFO brace
Patient 3	23	M	GSW to lower thigh	End-to-side	6 mo	Assisted- walker	0	Absent in CPN distribution	Allograft ×2	4 cm, 3 cm	8 mo	5	Walks unassisted
Patient 4	50	M	Knee fracture dislocation	End-to-side	15 mo	Assisted- AFO brace	0	Decreased in CPN distribution	Allograft ×2	5 cm, 17 cm	3 y	2	Walks unassisted

Abbreviations: AFO, ankle-foot-orthosis; F, female; GSW, gunshot wound; M, male; MRC, Medical Research Council; N/A, not available.

## Discussion

When the nerve is not amenable to direct repair or grafting, options for repair include the traditional end-to-end nerve transfer, end-to-side nerve transfer, and less commonly, the side-to-side transfer. Current techniques in nerve repair have inconsistent results, and the wide variety of options makes it challenging to identify a clear superiority of any one strategy at this time. 16 17

Despite overall inconsistencies in results across techniques, some authors maintain that the traditional end-to-end style of coaptation cannot be replaced entirely by end-to-side techniques by demonstrating a clinical advantage to the end-to-end techniques. 18 19 20 End-to-side techniques are typically favored in cases where the proximal stump or nerve donors are unavailable, thus cases where end-to-end repair is impossible. 21 Few studies have compared the differences between these two techniques. When comparing end-to-end versus end-to-side in the animal model, Liao et al noted that, although both techniques resulted in functional recovery, the end-to-end technique had faster recovery of nerve action potential response with more myelinated large fiber recovery. 19 Jaeger et al also reported that end-to-end had superior muscle mass preservation. 18

Interestingly, Jaeger et al also reported that both had similar muscular atrophy while sensory results increased recovery discrepancy when comparing the two techniques. 18 Furthermore, Cederna et al reported that end-to-side had higher denervated muscle fibers although not affecting donor muscle. 14 Liao et al suggested that these differences between repairs are due to an end-to-end repair being mediated by the regeneration of severed axons while end-to-side is through a collateral regeneration of donor axons. 19

Although these articles present a comparison of techniques, it is essential to note that they do not always use the same donor and recipient nerves across end-to-end and end-to-side groups. Specifically, two articles in solid support of the end-to-end technique, Liao et al and Jaeger et al used different donor nerves in the end-to-end cases than in their end-to-side cases, making it hard to interpret these results. 18 19

Much like the cross-bridge technique described in this article, the direct side-to-side neurorrhaphy benefits from the fact that transection of neither the donor nor recipient's nerves is required, potentially sparing donor nerve function, as well as allowing axonal regeneration through the distal end of the recipient nerve overtime. This technique has been explored in animal models and found to be protective against significant muscle atrophy and can lead to improved functional outcomes. 16

The cross-bridging strategy has shown potential success in various animal studies. In 2011, Ladak et al demonstrated in rats that cross-bridging with nerve grafts between donor tibial nerves and recipient peroneal nerves in an end-to-side manner was protective against the distal effects of chronic denervation and that the level of protection increased with the number of cross-bridges created, resulting in higher numbers of peroneal nerve axons regenerating from the intact proximal nerve to the denervated distal stump, as well as increased end-organ muscle weights. 8 Similar results were demonstrated by Gordon et al in 2015 in a rat model of delayed nerve repair. 7 The main advantage of this technique is minimizing both donor and recipient nerve trauma. Traditional end-to-end or end-to-side nerve transfers require either partial or total transection of the donor's nerve; however, this cross-bridging technique requires only tiny perineurial windows in the donor and recipient nerves.

Our study demonstrates clinical recovery of nerve function in four patients who had severe, persistent nerve injury using the cross-bridging ladder technique with end-to-side neurorrhaphies. The patients who underwent surgery with our technique showed functional improvement for months to years from MRC of 0 to an MRC of 2 to 5. Early intervention (within 6 months of injury) yielded the best outcome from our cases. This is expected as more recent investigations have demonstrated that early reinnervation of the distal nerves and their end organs can slow the onset of these chronic denervation-related changes. 22 In three out of the four patients, there was an early improvement from MRC of 0 to 2 and continued recovery in patient 3, which demonstrates that this technique can allow nerve regeneration and some regain of function within months of surgery, possibly due to the number of cross-bridge allowing for a higher number of nerve axon regeneration through the donor nerve sprouting axons into to the denervated recipient nerve via the cross-bridges. 6 23 Patient 4, although not having much improvement in the earlier recovery period, had an MRC of 2 after 1.5 years showing that delayed nerve recovery is also possible with this technique.

## Limitations

This study has several limitations, most notable being the small sample size of only four patients and one surgeon (senior author). Follow-up is relatively short in two of the four patients. Additionally, while the MRC grading system is theoretically a standardized method of measuring muscle strength, practically, it continues to have the potential for subjectivity in its use. Therefore, more extensive prospective clinical studies are needed to confirm this cross-bridge ladder technique's success in treating CPN dysfunction. Lastly, this is a case series without a control group to assess the effectiveness of the technique on the recovery of the CPN dysfunction.

## Conclusion

Overall, we demonstrate the utility and clinical outcomes of the cross-bridge ladder technique (H-shaped) in patients with persistent and prolonged foot drop following trauma. However, recovery was varied in patients; all regained motor function, with some patients improving after the most recent follow-up. Larger series are needed to demonstrate the validity of this technique, especially when adopted by other surgeons in different institutions, to look for reproducibility of the results.

## References

[1] Li H Terenghi G Hall S M Effects of delayed re-innervation on the expression of c-erbB receptors by chronically denervated rat Schwann cells in vivo Glia 1997 20 04 333 347 9262237 10.1002/(sici)1098-1136(199708)20:4<333::aid-glia6>3.0.co;2-6

[2] Li H Wigley C Hall S M Chronically denervated rat Schwann cells respond to GGF in vitro Glia 1998 24 03 290 303 9775980

[3] Fu S Y Gordon T Contributing factors to poor functional recovery after delayed nerve repair: prolonged axotomy J Neurosci 1995 15(5 Pt 2):3876 3885 7751952 10.1523/JNEUROSCI.15-05-03876.1995 PMC6578210

[4] Fu S Y Gordon T Contributing factors to poor functional recovery after delayed nerve repair: prolonged denervation J Neurosci 1995 15(5 Pt 2):3886 3895 7751953 10.1523/JNEUROSCI.15-05-03886.1995 PMC6578254

[5] Verdú E Ceballos D Vilches J J Navarro X Influence of aging on peripheral nerve function and regeneration J Peripher Nerv Syst 2000 5 04 191 208 11151980 10.1046/j.1529-8027.2000.00026.x

[6] Koliatsos V E Price W L Pardo C A Price D L Ventral root avulsion: an experimental model of death of adult motor neurons J Comp Neurol 1994 342 01 35 44 8207127 10.1002/cne.903420105

[7] Gordon T Hendry M Lafontaine C A Cartar H Zhang J J Borschel G H Nerve cross-bridging to enhance nerve regeneration in a rat model of delayed nerve repair PLoS One 2015 10 05 e0127397 26016986 10.1371/journal.pone.0127397 PMC4446033

[8] Ladak A Schembri P Olson J Udina E Tyreman N Gordon T Side-to-side nerve grafts sustain chronically denervated peripheral nerve pathways during axon regeneration and result in improved functional reinnervation Neurosurgery 2011 68 06 1654 1665, discussion 1665–1666 21346654 10.1227/NEU.0b013e31821246a8

[9] Vuorinen V Siironen J Röyttä M Axonal regeneration into chronically denervated distal stump. 1. Electron microscope studies Acta Neuropathol 1995 89 03 209 218 7754742 10.1007/BF00309336

[10] You S Petrov T Chung P H Gordon T The expression of the low affinity nerve growth factor receptor in long-term denervated Schwann cells Glia 1997 20 02 87 100 9179594 10.1002/(sici)1098-1136(199706)20:2<87::aid-glia1>3.0.co;2-1

[11] Höke A Gordon T Zochodne D W Sulaiman O A A decline in glial cell-line-derived neurotrophic factor expression is associated with impaired regeneration after long-term Schwann cell denervation Exp Neurol 2002 173 01 77 85 11771940 10.1006/exnr.2001.7826

[12] Sulaiman O A Gordon T Effects of short- and long-term Schwann cell denervation on peripheral nerve regeneration, myelination, and size Glia 2000 32 03 234 246 11102965 10.1002/1098-1136(200012)32:3<234::aid-glia40>3.0.co;2-3

[13] Sulaiman O A Gordon T Role of chronic Schwann cell denervation in poor functional recovery after nerve injuries and experimental strategies to combat it Neurosurgery 2009 65(4, Suppl):A105 A114 19927054 10.1227/01.NEU.0000358537.30354.63

[14] Cederna P S Kalliainen L K Urbanchek M G Rovak J M Kuzon W M Jr “Donor” muscle structure and function after end-to-side neurorrhaphy Plast Reconstr Surg 2001 107 03 789 796 11310430 10.1097/00006534-200103000-00021

[15] Lundborg G Structure and function of the intraneural microvessels as related to trauma, edema formation, and nerve function J Bone Joint Surg Am 1975 57 07 938 948 171272

[16] Rönkkö H Göransson H Taskinen H S Paavilainen P Vahlberg T Röyttä M Protective distal side-to-side neurorrhaphy in proximal nerve injury-an experimental study with rats Acta Neurochir (Wien) 2019 161 04 645 656 30746570 10.1007/s00701-019-03835-2 PMC6431300

[17] Giuffre J L Bishop A T Spinner R J Levy B A Shin A Y Partial tibial nerve transfer to the tibialis anterior motor branch to treat peroneal nerve injury after knee trauma Clin Orthop Relat Res 2012 470 03 779 790 21626085 10.1007/s11999-011-1924-9 PMC3270157

[18] Jaeger M R Braga-Silva J Gehlen D End-to-end versus end-to-side motor and sensory neurorrhaphy in the repair of the acute muscle denervation Ann Plast Surg 2011 67 04 391 396 21587041 10.1097/SAP.0b013e3182126816

[19] Liao W C Chen J R Wang Y J Tseng G F The efficacy of end-to-end and end-to-side nerve repair (neurorrhaphy) in the rat brachial plexus J Anat 2009 215 05 506 521 19682138 10.1111/j.1469-7580.2009.01135.x PMC2780569

[20] Spyropoulou G A Lykoudis E G Batistatou A New pure motor nerve experimental model for the comparative study between end-to-end and end-to-side neurorrhaphy in free muscle flap neurotization J Reconstr Microsurg 2007 23 07 391 398 17975766 10.1055/s-2007-992347

[21] Sulaiman O AR Gordon T A rat study of the use of end-to-side peripheral nerve repair as a “babysitting” technique to reduce the deleterious effect of chronic denervation J Neurosurg 2018 131 02 622 632 30215557 10.3171/2018.3.JNS172357

[22] Tos P Colzani G Ciclamini D Titolo P Pugliese P Artiaco S Clinical applications of end-to-side neurorrhaphy: an update BioMed Res Int 2014 2014 646128 25136607 10.1155/2014/646128 PMC4127263

[23] Viterbo F Trindade J C Hoshino K Mazzoni A Two end-to-side neurorrhaphies and nerve graft with removal of the epineural sheath: experimental study in rats Br J Plast Surg 1994 47 02 75 80 8149062 10.1016/0007-1226(94)90162-7

